# Globally Accessible Distributed Data Sharing (GADDS): a decentralized FAIR platform to facilitate data sharing in the life sciences

**DOI:** 10.1093/bioinformatics/btac362

**Published:** 2022-05-27

**Authors:** Pavel Vazquez, Kayoko Hirayama-Shoji, Steffen Novik, Stefan Krauss, Simon Rayner

**Affiliations:** Hybrid Technology Hub – Centre of Excellence, Faculty of Medicine, Institute of Basic Medical Sciences, University of Oslo, Oslo 0317, Norway; Hybrid Technology Hub – Centre of Excellence, Faculty of Medicine, Institute of Basic Medical Sciences, University of Oslo, Oslo 0317, Norway; Department of Informatics, Faculty of Mathematics and Natural Sciences, University of Oslo, Oslo 0315, Norway; Hybrid Technology Hub – Centre of Excellence, Faculty of Medicine, Institute of Basic Medical Sciences, University of Oslo, Oslo 0317, Norway; Department of Immunology and Transfusion Medicine, Oslo University Hospital, Oslo 0424, Norway; Hybrid Technology Hub – Centre of Excellence, Faculty of Medicine, Institute of Basic Medical Sciences, University of Oslo, Oslo 0317, Norway; Department of Medical Genetics, Oslo University Hospital, Oslo, 0407, Norway

## Abstract

**Motivation:**

Technical advances have revolutionized the life sciences and researchers commonly face challenges associated with handling large amounts of heterogeneous digital data. The Findable, Accessible, Interoperable and Reusable (FAIR) principles provide a framework to support effective data management. However, implementing this framework is beyond the means of most researchers in terms of resources and expertise, requiring awareness of metadata, policies, community agreements and other factors such as vocabularies and ontologies.

**Results:**

We have developed the Globally Accessible Distributed Data Sharing (GADDS) platform to facilitate FAIR-like data-sharing in cross-disciplinary research collaborations. The platform consists of (i) a blockchain-based metadata quality control system, (ii) a private cloud-like storage system and (iii) a version control system. GADDS is built with containerized technologies, providing minimal hardware standards and easing scalability, and offers decentralized trust via transparency of metadata, facilitating data exchange and collaboration. As a use case, we provide an example implementation in engineered living material technology within the Hybrid Technology Hub at the University of Oslo.

**Availability and implementation:**

Demo version available at https://github.com/pavelvazquez/GADDS.

**Supplementary information:**

Supplementary data are available at *Bioinformatics* online.

## 1 Introduction

The advances in experimental methodologies in the past two decades have produced a revolution in the life sciences (Cohen-Boulakia *et al.*, 2017). Researchers not only generate huge amounts of data in a single experiment but also the types of data they collect have become highly divergent. Consequently, the discipline is acquiring data science methodologies and adopting a ‘life cycle’ view of research data (Djordjevic *et al.*, 2019), researchers are now facing the challenges associated with handling large amounts of heterogeneous data in a digital format. Some of these challenges include: consolidating heterogeneous data; translating data into a format that can be read by complex analysis pipelines; determining the most suitable analysis parameters; and ensuring data provenance for full reproducibility (Gruning *et al.*, 2018). Also, as life science research increasingly occurs in multidisciplinary and interactive environments, data need to be easily accessible among collaborators while allowing owners to retain a level of control. Existing solutions, where data are collected and stored in a centralized resource, are not necessarily ideally suited to such collaborative environments and users can benefit from federated solutions where data are distributed across a shared infrastructure.

Traditionally, data sharing solutions in the life sciences have adopted a centralized architecture to serve up a single data type. For example, the Sequence Read Archive (SRA) (Kodama *et al.*, 2012) provides a public repository for next-generation sequence data. In recognition of the need to integrate heterogeneous data types, tools such as InterMine (Smith *et al.*, 2012) allow users to query and analyse integrated yet diverse datasets but implementing InterMine for personal research needs is challenging. In addition, a centralized solution is dependent on the continued participation of the partner hosting the resource, which may be dependent on factors such as continued funding (Imker, 2018). In contrast, a decentralized solution shares responsibility among all participants so that new members can be added, and the resource endures when participants depart. Currently, the most common decentralized solutions used in the life sciences involves private third-party solutions, such as Dropbox (https://www.dropbox.com/, Last access: March, 22, 2022) or OneDrive (https://onedrive.live.com/, Last access: March, 22, 2022). These solutions allow the user to retrieve previous data versions which is a desirable feature for many researchers. However, these platforms can compromise privacy and offer limited control of data access (Alotaibi *et al.*, 2019).

There is also growing evidence to suggest that many published results will not be reproducible over time (Curty *et al.*, 2017; Vines *et al.*, 2014) and robust data management and stewardship plans are needed to ensure data sustainability (Eisenstein, 2022; Griffin *et al.*, 2017). Thus, there is a need for data descriptors, i.e. metadata, to describe the raw data that are collected as part of an experiment. For example, to reproduce a next-generation sequencing experiment or to compare results with experiments from other studies, the biological material and NGS experimental conditions such as library prep and adapter information are needed (Chiara *et al.*, 2021; Zhong *et al.*, 2019). To this end, the SRA requires metadata to accompany data submission to collect key experimental information. This includes information such as *instrument* (e.g. Illumina versus Nanopore), *Assay* (e.g. Small RNA or RNA seq) and *Sample Type* (Tissue or Cell Line).

Thus, good practice requires researchers to store both the metadata and data or **(meta)data** for a single data instance, as a standard step in a study. An early example of a basic implemented descriptive metadata template was the Dublin core standard (Board, 2014). The Dublin metadata core elements consist of: *Creator*, *Contributor*, *Publisher*, *Title*, *Date*, *Language*, *Format*, *Subject*, *Description*, *Identifier*, *Relation*, *Source*, *Type*, *Coverage* and *Rights*.

To aid reproducibility efforts, the Findable, Accessible, Interoperable, Reusable (FAIR) data initiative was introduced to provide a framework for defining the minimum elements required for good data management (Wilkinson *et al.*, 2016). As publishers, funding agencies and policymakers are becoming increasingly aware of the FAIR data initiative there have been efforts to implement measurable indicators of FAIRness in scientific research (Wilkinson *et al.*, 2019). Some of the findings indicate that current data management solutions and communities only achieve a partial level of FAIRness. Moreover, the lack of exactness in the original FAIR principles means that there are no clear implementation guidelines. Thus, there is no clear path to achieving full standardization and mechanisms to control the metadata quality are needed (Koers *et al.*, 2020). For instance, the SRA metadata is only partially verified (Bernstein *et al.*, 2017; Zhu *et al.*, 2013). In many cases, key information regarding library and adapter information, which is necessary for processing raw data, is missing (Zhong *et al.*, 2019).

Although the importance of FAIR has been recognized widely by the research community via initiatives such as GO-FAIR (https://www.go-fair.org/, Last access: March, 22, 2022), implementing these recommendations are beyond the means of most researchers in terms of both resources and expertise (Barone *et al.*, 2017; Cohen-Boulakia *et al.*, 2017; Fillinger *et al.*, 2019). First, to create an effective data plan, users need to become data literate (Gray *et al.*, 2018) by acquiring an understanding of metadata, schemata (McQuilton *et al.*, 2016), protocols, policies (Thessen and Patterson, 2011), annotations (Osumi-Sutherland *et al.*, 2021) and community agreements (Cox *et al.*, 2021; Sansone *et al.*, 2019). Second, when robust solutions exist (McQuilton *et al.*, 2016; Smith *et al.*, 2012; Tekle *et al.*, 2018), users may have to choose among different and not necessarily compatible realizations. Finally, users must also implement their data plan as a software/hardware solution. Software such as COPO (Shaw *et al.*, 2020) have taken a first step to provide off the shelf solutions, allowing users to set up a data server that can describe research data using community sanctioned vocabularies. Using COPO, individual research groups can make their annotated data available to the research community, or research collaborations can share their data in a standardized way. However, COPO uses a traditional centralized data architecture.

In summary, while there is clear recognition of the need for (i) FAIR compliance and (ii) user-friendly distributed and scalable data sharing solutions which (iii) allow users to retain control of their data, there are limited options available. Current solutions only address one or two of these requirements. Consequently, responding to the rapidly evolving data universe and developing new solutions to address specific needs in data collection while achieving FAIRness represents a major challenge for many researchers in the life sciences.

**Fig. 1.**
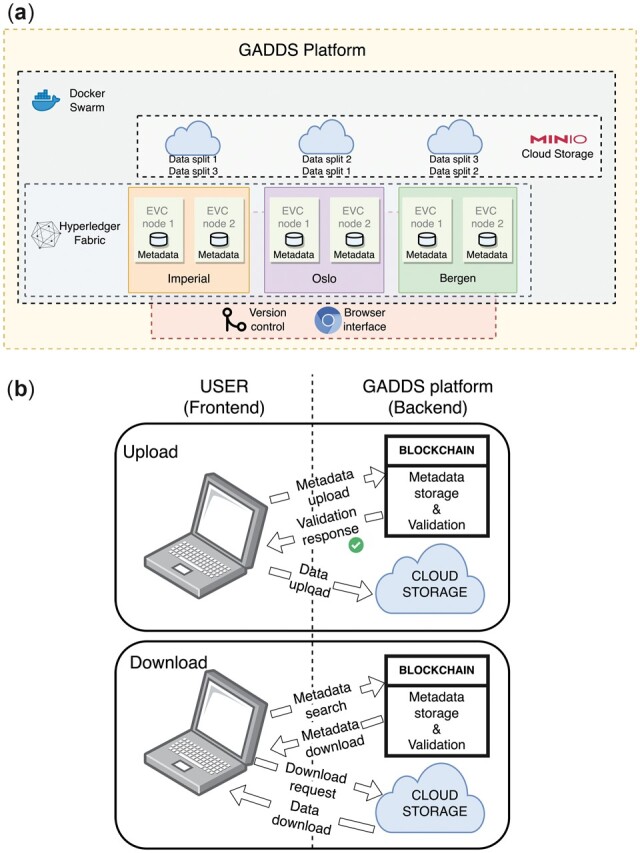
Schematic of a basic implementation of the GADDS platform. (**a**) Architecture. The platform is deployed as a Docker Swarm cluster. The schematic shows a platform architecture distributed across three organizations (Imperial College London; Oslo University Hospital, Oslo; and University of Bergen, Bergen). Each organization hosts a blockchain service provided by Hyperledger Fabric with two peers (EVC nodes) each of which are responsible for metadata validation (to ensure quality control) and metadata storage. The corresponding data are stored on a privately maintained cloud provided by MinIO, where data are split and replicated across the storage infrastructure. The version control system is provided by the Distributed Version Control (DIVECO) software and secure access to the platform is through a web browser. (**b**) User interaction. The user interacts with the GADDS platform through the web browser. During the upload, metadata is submitted for storage and validation to the blockchain service using a simple form. After the metadata is validated, the user is allowed to upload data to the cloud. During the download, the user searches the blockchain’s ledger for stored and validated metadata; once the metadata is retrieved, the user is able to download the associated data

We have developed the Globally Accessible Distributed Data Sharing (GADDS) platform, an all-in-one scalable cloud environment to facilitate data archiving and sharing in research collaborations while offering a level of FAIRness. A schematic is shown in Figure 1. The GADDS platform uses decentralization technologies and a tamper proof blockchain algorithm to enforce metadata quality control. A cloud architecture storage system is used where a data object is split, replicated and stored across multiple devices. Version control software was developed to handle (meta)data submits and changes/updates. A web browser-based interface is provided to simplify the data collection, data storage and data retrieval processes for the end user.

In this work, GADDS was implemented for fiber cell tissue research, a component of engineered living materials (ELM) technology, an emerging field in need of FAIR-based data solutions (Wu *et al.*, 2020). However, the GADDS platform has general application in any data collection and integration process requiring a level of data FAIRness. In this article, we present the motivation, conceptualization and the architecture of the platform and demonstrate how it facilitates data archiving and sharing with a level of FAIRnes*s*

## 2 Systems and methods

The GADDS platform is motivated by two data paradigms—data decentralization and FAIR data. We have used blockchain and cloud technologies to procure decentralization, while a level of FAIRness is reached through openness of metadata and using minimum standards. The advantages of using blockchain technology are: first, it implements decentralized decision-making (in our case, the metadata validation) performed by independent nodes. This enhances security as there is not a single entity deciding which metadata is valid. Second, the ledger (in our case, the metadata database) is open and replicated on each computer. This means that the metadata is freely accessible to everyone that has access to the GADDS platform but any tampering in the ledger (i.e. unintended modifications that are not consistent with the predefined standard) will result in identifiable inconsistencies.

The GADDS also tracks changes in metadata. Metadata is permanently stored in the ledger, thus if newer versions of metadata (modifications) are submitted then the new metadata is linked to the same data while a history of changes is retained.

Collaboration between research groups often occurs across institutions, thus it is common to find infrastructure differences (e.g. researchers might work though virtual machines (VMs) instead of physical computers), but these differences can be minimalized through containerization.

### 2.1 Architectural details

The GADDS platform’s architecture is geographically distributed, where hardware resources are a collective of participants (e.g. research institutes or universities), this type of architecture allows the platform to continue to perform its function and oversee metadata quality assurance in the event of hardware failure. For example, in small-scale research collaborations, implementing centralized RAID technology usually places the onus on a single user. With the GADDS platform, responsibility is shared, if nodes go down (e.g. in places with sporadic internet) the system continues functioning and resynchronization occurs when connectivity is restored.

The GADDS platform is a hybrid of three technologies: blockchain for decentralization and fault-tolerance in the quality control system, cloud object storage for distributed fault-tolerant data storage and a versioning system to track changes in data. In the following sections, we describe these three components in more detail.

We use Docker containers and Swarm technology for its ease of use and flexibility. Full architecture details of the GADDS platform are provided in the Supplementary Materials. A key component of the architecture are the Endorser, Validator and Committer (**EVC**) nodes (see below). In the current GADDS platform implementation, we have simplified the allocation of containers, such that an organization accommodates two **EVC** nodes, an **Orderer**, a Web Interface and a Cloud Storage. Nevertheless, Docker flexibility allows different configurations where containers can be allocated using different hardware.

### 2.2 Blockchain

Blockchain is the backbone of the Bitcoin cryptocurrency (Nakamoto, 2019) but is also well suited to more general data tracking tasks. Blockchain is a decentralized database and decision maker that records all transactions (exchanges) that have been executed among participants. The decision-making is invoked through a validation algorithm where transactions must be endorsed by the majority of participants (nodes) of the system. The database, called the ledger, contains every single record of each transaction in the form of blocks. In the GADDS platform, a transaction is the validated metadata and its key features are:

- Decentralization: the open ledger is shared and participants must agree that a transaction is valid.
- Security: access to the blockchain is through permissions and metadata is cryptographically signed.
- Transparency: every node in the blockchain has a copy of the ledger.

In the GADDS platform, the consensus is an agreement about whether to include specific metadata into the ledger, Supplementary Figure S1 shows the full details of the (meta)data lifecycle. Essentially, the consensus ensures that every new block that is added to the ledger is the one and only version that is agreed upon by the majority of participants. In this way, the consensus algorithm establishes trust between peers in a distributed computing environment.

In the GADDS platform, we use the Hyperledger Fabric Proof-of-Authority (PoA) Byzantine-based consensus protocol (Cachin, 2016;Supplementary Fig. S2). The advantage of using PoA is that transactions, in our case metadata validations, are not computationally demanding, requiring less energy consumption and the rate of validations is much faster compared to Proof of Work (PoW). Both PoA and PoW have a high-risk tolerance, as long as 51% of the endorser-validators are available or not acting maliciously.

In the GADDS platform, the blockchain plays a dual role of performing metadata quality control and acting as a database for the metadata. Here, the PoA consensus algorithm uses Hyperledger Fabric to validate the presented metadata to ensure that is following a predefined standard (see User Case). The Hyperledger Fabric uses dedicated nodes called peers, they act as endorsers, validators and committers, and we collectively refer to these nodes as EVCs. These EVCs allow organizations to collaborate in the validation of metadata and the formation of blocks. Consistent with the Hyperledger Fabric standard, the GADDS platform performs these three functions within single dedicated nodes, while the Orderer function is assigned to separate nodes. Thus, the EVC nodes perform validation of the metadata, while the Orderer performs ordering of the metadata and packaging into a block, for more information see Supplementary Materials.

### 2.3 Data storage

Rather than using dedicated servers offering traditional networked data storage, the GADDS platform uses a cloud architecture where data are split, replicated and stored across multiple devices. This helps to protect the data against failure events such as corruption, hardware failure or intrusions. This approach offers a high level of redundancy as it is possible to lose up to half (N/2) of the total storage devices and still recover the data, while the storage technology ensures that end users see data represented as a single file on the server.

The GADDS platform implements MinIO (https://www.min.io/, Last access: April 5, 2022) for cloud storage technology (Lin and Tzeng, 2011). We selected MinIO as it is open source, relatively straightforward to deploy through a Docker swarm and is well documented and supported. In the GADDS platform, each part of an object is distributed across multiple secure organizations (Supplementary Fig. S3), each of which corresponds to a physical location in a separated secure environment running a dedicated MinIO Docker container.

### 2.4 Version control

As part of the distributed design of the GADDS platform, a version control software, DIVECO, was developed to work with the MinIO storage solution to handle (meta)data submits and changes/updates. In this way, projects can scale up to handle large numbers of participants for geographically distributed research. DIVECO is capable of recording changes made to (meta)data entries, so that submissions that have been already validated can be modified. When submitting a change to the (meta)data through the web interface, the process of validation is initiated as if it was a new entry. If the validation is successful, a new block of metadata is created. The older version of the metadata is retained so, consistent with other version control software such as Git (Git, 2021), it is possible to ‘go back in time’ to retrieve a specific version of that metadata. However, the corresponding data cannot be modified, thus both versions of the metadata point to the same data (Supplementary Fig. S4).

## 3 Results

The GADDS platform is designed to be deployed as a geographically distributed enterprise to aid the sharing of standardized data among laboratories and institutions implementing diverse technologies but working toward a common goal. The platform is a global federation (i.e. a group of computing resources sharing similar standards) where instances of resources form a unified global namespace. In this way, the GADDS platform is theoretically able to support an unlimited number of distributed participants.

A schematic of a test implementation of the GADDS platform is shown in Figure 1. The platform is configured to be a Docker swarm cluster of a group of physical or virtual **machines** which execute well defined tasks as applications, and **nodes** which are machines that have been configured to be joined together in a network. Following the Hyperledger Fabric architecture definitions, an **organization** consists of one or more nodes that share the same **domain name**. A **channel** is a *permissioned* network through which different organizations communicate, and organizations that share a common channel form a **consortium**. Organizations within a consortium share a unique ledger. The Hyperledger Fabric has the flexibility to adopt different organization, channel and consortium configurations; in this way, data can be shared in a secure manner but be physically distinct so that a catastrophic event, such as a server failure or an intrusion, remains an isolated event.

In the GADDS implementation presented in this article, we have configured three Hyperledger Fabric organizations in three different locations: Imperial College London, Oslo University Hospital and the Norwegian Research and Education Cloud [NREC (https://www.nrec.no/, Last access: April 5, 2022)] located in Bergen. This is shown schematically in Figure 1, with the three organizations being placed within a single consortium and communicating via a single channel. For simplicity, we have configured all nodes to be Docker Swarm managers. The MinIO data storage and the blockchain Hyperledger Fabric are instantiated within the Docker environment. DIVECO and the browser interface are also within the Docker environment as a single separated container.

As the GADDS platform is a permissioned environment, only users from the same consortium can download data. In our example, all organizations belong to a single consortium, thus all participants can share the data. Metadata and data are stored separately within each organization. EVC nodes participate in the blockchain and store the open metadata, while other assigned nodes (i.e. storage machines or servers) in the organizations store the data in a secure environment. A more detailed discussion about the blockchain functionalities is presented in Supplementary Materials.

We have made the GADDS platform highly configurable. It is possible to add users who are allowed to submit (meta)data to the system. At the same time, it is possible to use channels to create collaborations, with different configurations of users working on different projects and datasets. Also, due to the use of containerized technologies, the platform can be readily scaled, so that new blockchain participants (EVC nodes) or new storage servers can easily be added. Thus, local groups (organizations) of storage servers and EVC nodes can be configured in specific geographical locations with their own local infrastructure. It is also possible to have the GADDS platform locally installed but still connected to other installations (i.e. to share ledgers and contribute to cloud storage) in different locations without losing local performance (i.e. data retrieval/submission due to connectivity problems). At the same time, local installations typically have local security implementations, which helps to keep failures or intrusions in isolated parts, so an issue with one organization does not affect the entire system.

### 3.1 FAIR

The GADDS platform uses blockchain as a novel form to achieve a level of FAIRness. The FAIR initiative is a guideline to help data to be Findable, Accessible, Interoperable and Reusable. The principle of **Findability** stipulates that data should be identified, described and registered or indexed in a clear and unequivocal manner; in the GADDS platform, we have made it a requisite that data must be described with relevant metadata while using unique internal identifiers, aiding searchability. The principle of **Accessibility** proposes that datasets should be available through a clearly defined access procedure, ideally by automated means; in the GADDS platform, the data access process is clearly defined, but incorporated within the web browser interface to ease the user experience. Thus, meta(data) is open and free, subject to a process of authentication and authorization. The principle of **Interoperability** recommends that data and metadata are conceptualized, expressed and structured using common standards; metadata stored in the GADDS platform uses clear predefined standards and vocabularies, using shared elements from standard vocabularies to describe a feature (such as cell type), then it is possible to relate data from different sources. **Reusability** is the ultimate aim of FAIR, and it is proposed that this should be achieved via the use of well-described metadata and data according to domain relevant community standards. The GADDS platform can accommodate any metadata standard and it enforces the adoption of these standards when submitting data. Metadata and its matching data are stored as a duple, metadata ‘points’ to the data, facilitating data reuse.

### 3.2 Use case

**Fig. 2.**
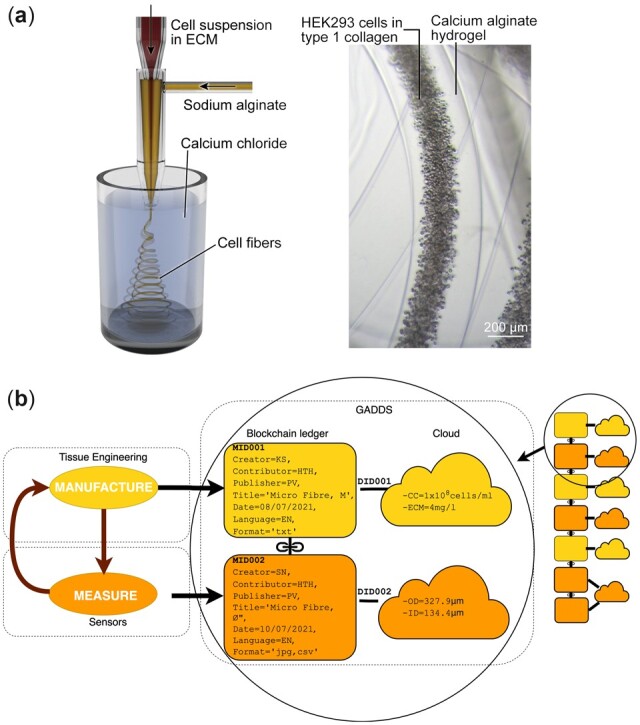
Schematic of a basic implementation of the GADDS platform. (**a**) Architecture example. Manufacture and measurement of microfibres. Experiments were conducted by the Tissue Engineering Group in the Hybrid Technology Hub, University of Oslo (UiO) and the Sensors group in the Department of Physics at UiO. (a) *left*: *schematic of fiber manufacturing process*. Fibers are formed by injecting a solution of HEK293 cells and bovine collagen through the central channel of a co-axial glass capillary nozzle, mixing with sodium alginate from the outer channel, and injecting into calcium chloride solution for crosslinking. After core-shell hydro-fibers are formed, they are transferred to culture medium and incubated. *Right*: *microscope image of generated fibers*. Generated fibers were characterized by measurement of inner and outer diameter at three points along the axis. (**b**) Schematic showing data collection and storage in the GADDS platform. *Left*: Manufacture and Measurement is performed in an iterative manner to determine relationships between experimental and measured parameters. *Middle*: Meta(data) upload to GADDS. Metadata is verified and stored in the ledger, and corresponding experimental data are stored in the cloud. Metadata and data are linked by a unique Data ID (DID). *Right*: successive measurements are added to the ledger. The bottom of the ledger shows a case where metadata has been modified, but still points to the same data

To demonstrate the use of the GADDS platform in a scientific environment from an end user perspective (Supplementary Fig. S5), we generated and processed (meta)data from a series of experiments to produce tissue fibers. This was conducted by the Tissue Engineering group within the Hybrid Technology Hub (https://www.med.uio.no/hth/english/, Last access: April 5, 2022) and the Sensors group in the Department of Physics at UiO. The cell fibers are the functional tissue in fiber shape, encapsulated in thin alginate calcium hydrogel and, as a base unit of major organs, such as skeletal muscle, blood vessels and neurons, they have a wide range of applications such as implants and regenerative medicine (Onoe *et al.*, 2013). Figure 2a shows a schematic of the experiment. Briefly, the experiment involves two phases: *Phase 1* (Manufacture) involves fabrication of a core–shell microfiber and *Phase 2* (Measurement) involves characterization of the generated fibers using optical microscopy. Based on the microscopy data, the fabrication process can be revised, repeated and recharacterized, and the process repeated in an iterative manner.

*Phase 1. Tissue Engineering (Fiber Manufacture)*: The production of the core–shell hydrogel fibers followed the procedures described in detail elsewhere (Onoe *et al.*, 2013) and consisted of the following major steps. The fibers were formed using a glass capillary co-axial nozzle. The core channel solution consisted of HEK293 cells mixed with 4 mg/ml of type 1 bovine collagen at a concentration of 1 × 10^8^ cells/ml. Sodium alginate solution (KIMICA, I-1G; 1 wt%) was infused from the outer channel at 200 µl/min to form the peripheral flow. The co-axial flow was ejected into 100 mM calcium chloride solution to crosslink the alginate, and hydrogel fibers were extruded from the outlet of the nozzle. After the formation of the fibers, they were immediately transferred into culture medium and incubated at 37°C for cell culturing. *Phase 2. Sensors (Fiber Measurement):* In the second phase, manufactured fibers were characterized by measuring the diameter of the outer shell and inner core at three points along the fibers using at least five optical microscope images (Fig. 2a right).

As part of the (meta)data entry process, see Figure 2b, the information was divided into metadata and data. We applied the concepts of data and metadata throughout as described by the *FAIR guiding principles* original article (Wilkinson *et al.*, 2016): *Metadata* *is* *any description of a resource that can serve the purpose of enabling findability and/or reusability and/or interpretation and/or assessment of that resource* (i.e. metadata is the descriptor, and data are the thing being described).

Since there is no relevant metadata standard specific to the fiber manufacture and the definition of metadata can differ among research communities, we defined our metadata template (i) to include descriptive metadata such as author, title and date and (ii) to enrich the metadata, we included experimental specific metadata that describes the internal structure of the data. Thus, different templates were created for the manufacture and measurement phases.

We then extended the Dublin core metadata elements to include relevant data descriptors within the ELM field. The structural metadata elements and the type of data collected for the experiments for *Phase 1* (Tissue Manufacture) and *Phase 2* (Fiber Measurement) are shown in Supplementary Tables S1 and S2, schemas for the metadata used are also included in the Supplementary Materials.

When uploading the dataset, the platform verifies through the metadata quality control system (blockchain) that the relevant metadata elements are defined according to the proper standard and that entries for all the elements are present, e.g. the language element must follow the ISO 639-1 standard of two letters.

## 4 Discussion

The increasing digitization of data is generating new possibilities in data analysis, data sharing and data interoperability. This is reflected in the many action plans created to support the development of FAIR data initiatives. However, implementing FAIR solutions are beyond the means of most researchers (Barone *et al.*, 2017; Cohen-Boulakia *et al.*, 2017; Fillinger *et al.*, 2019). There is a particular need for FAIR solutions that can (i) support distributed and controlled data sharing for individual researchers or within a research collaboration, (ii) be easily implemented and (iii) respond to a rapidly evolving data universe.

We have developed the GADDS platform which implements a distributed data management solution that offers open and well documented metadata while ensuring that predefined metadata standards are being followed. Thus, the GADDS platform helps researchers achieve a basic level of FAIRness in their data management. The platform can be downloaded and configured across a network of nodes at different physical locations using a series of setup scripts. By using containerized technology, the GADDS platform can be readily scaled, so that new organizations or new storage servers can easily be added, and participants can be removed without impacting the longevity of the data. Additionally, local groups of storage servers and nodes can be configured in specific geographical locations with their own local infrastructure. In this way, a local infrastructure has its own security implementations, which helps to isolate failures or intrusions, so an issue with one organization does not affect the entire system.

The GADDS platform has the following key features.

1. The platform uses blockchain technology that works as an open database and as a metadata quality control to guarantee compliance with predefined metadata standards. In the example implementation of the GADDS platform, we extended the predefined Dublin metadata standard and future versions will allow researchers to implement their own standards. Thus, the GADDS platform will be able to incorporate metadata standards from repositories such as FAIRsharing (Sansone *et al.*, 2019). In this way, users can easily select their own standards to describe their data, or core user groups can use the GADDS platform to develop a common set of standards for their specific research topic.
2. Metadata is cryptologically stored in the blockchain’s ledger, this gives researchers clear data ownership and recognition (Chevet, 2018). This is similar to efforts such as artifacts.ai (https://artifacts.ai/, Last access: April 5, 2022) which aims to link researchers with their research output via a blockchain ledger. The GADDS platform has the potential to support and integrate with such recognition efforts, for example, through integration of researchers’ ORCIDs (https://orcid.org/, Last access: April 5, 2022).
3. The platform is decentralized. Data are stored in drives that are distributed across several nodes and it resist multiple node failures, while ensuring recovery and enhanced security.

The metadata standards used in a GADDS platform implementation are predefined and their development is commonly part of a community effort. For example, in the ELM community, FAIR concepts have yet to be incorporated and currently no environment exists to aid the development of standards; in the GADDS platform such standards can be easily implemented and tested on a community wide basis, and then revised and updated.

The GADDS platform is not intended to store sensitive information (e.g. patient medical records). However, this could be accommodated by ensuring the metadata standards are designed so that sensitive information is only stored in the data associated with the entry in the ledger. For example, in the event of a patient withdrawing from a study, the sensitive information can then be permanently removed and the anonymized metadata relating to a removed patient will point to non-existing data. This approach has been previously proposed for handling remote access to Electronic Medical and Health Records (Dubovitskaya *et al.*, 2017; Rifi *et al.*, 2017).

In the presented implementation of the GADDS platform, metadata is accessible to participants through the blockchain ledger, but the data have restricted access (i.e. username and password are required). However, the (meta)data does not use a global unique identifier (such as a DOI) so it is possible that the created data silos cannot be used outside the GADDS environment and further steps might be needed to allow operation in a global capacity. Possible solutions include: implementation of secure identification mechanisms [such as the Feide (Melve and Solberg, 2007) authentication system used in Norway] or the application of individual persistent resolvable unique web identifiers (DOI) to the (meta)data duples. Alternatively, a public and open access ledger could be created. For example, to publish data associated with a research study, the relevant (meta)data could be exported from the private ledger to a dedicated open ledger. The metadata would not need to be revalidated against a standard template as this was already performed during the original submission. However, blockchain revalidation would be required to ensure data provenance as the order of the blocks will change (Supplementary Fig. S2). Future developments will also allow researchers to choose appropriate licencing and access characteristics to determine the level of openness of the data to further increase the reusability and interoperability.

While the GADDS platform facilitates distributed data sharing within research communities, it is not necessarily sufficient for translating data into a usable form. Governance measures and policies are also required, and research groups and/or institutions need dedicated bodies for data governance (Yebenes and Zorrilla, 2019). Finally, while the GADDS platform can help to simplify the process for data management, researchers need to make sense of their own data. Hence, data literacy is needed, and regular training of users and researchers remains a necessity (Koltay, 2017).

## Supplementary Material

btac362_Supplementary_Data
