# Supplementary material for: Globally Accessible Distributed Data Sharing (GADDS): a decentralized FAIR platform to facilitate data sharing in the life sciences
Source: Bioinformatics. 2022 May 27;38(15):3812–7. doi: 10.1093/bioinformatics/btac362 (PMC9344842; doi:10.1093/bioinformatics/btac362)
Supplement: btac362_Supplementary_Data [file btac362_supplementary_data.docx]

**Supplementary Materials:**

**Dictionary of Terms**

**(Meta)data**: data and metadata.

**Block:** sequence of submitted metadata entries.

**Bucket**: a basic *data container* in cloud storage (distinct from a *Docker container*).

**Channel**: permissioned network where *organizations* communicate.

**Cluster**: group of *machines*.

**Consortium**: group of *organizations* that share a common channel.

**Domain**: network address.

**EVC nodes:** same as peer, in the GADDS platform they act as **endorsers, validators** and **committers** (see methods).

**Experiment:** a **(**meta)data duple.

**GADDS**: Global Accessible Distribution Data Sharing.

**Ledger:** register that stores metadata in the blockchain.

**Machine**: physical hardware that can execute commands.

**Node**: *machine* in a network.

**OoC**: Organ-on-a-chip.

**Orderer:** packages and orders (meta)data duples into blocks.

**Organization**: group of *nodes* sharing a domain name.

**Project**: a collection of data and metadata duples.

**Transaction:** process where metadata is validated.

The following sections describe the details of specific aspects of the Global Accessible Distribution Data Sharing (GADDS) Platform.

1. **(Meta)data lifecycle**

The end users interact with the **EVC** nodes through a web interface where the data and metadata, or (meta)data, is separated. The data are stored in the cloud within **buckets**, while the metadata are stored in the ledger of the blockchain and assigned a unique Metadata Identifier (MID). The metadata and data remain linked by a unique Data Identifier (DID) to form a duple. A duple can also be considered an experiment, and a set of related experiments can form a project. The data from these experiments are placed inside the same bucket (identified with a unique project ID) within the cloud storage, *Supplementary Figure 1a*.

A schematic of the data lifecycle is shown in *Supplementary Figure 1b*. In the data upload step all three components (i.e. the blockchain, the cloud storage and the version control) of the GADDS platform participate. First the metadata need to be validated by the blockchain consensus algorithm (see next section) that operates among peers within the consortium (left hand side of *Supplementary Figure 1b*). Once the metadata are validated by consensus, it is incorporated into a **block** and is appended in the open ledger, i.e., the ledger is composed of a series of metadata in the form of blocks. At the same time, the data is uploaded as an object into a bucket in the cloud storage and a snapshot of the (meta)data with a timestamp is generated by the version control.


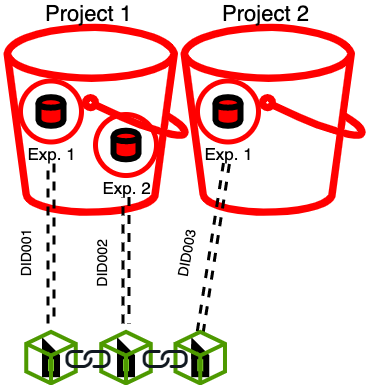


**(a)**


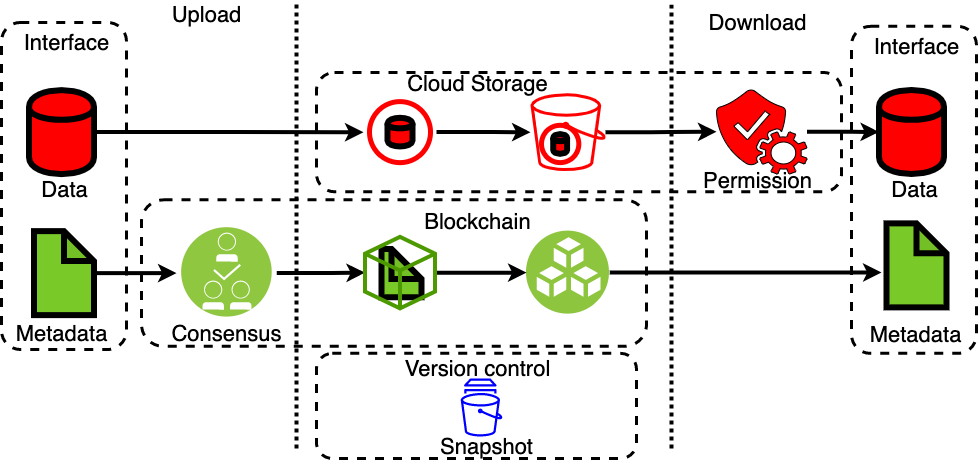


**(b)**

**Supplementary Figure 1. Overview of metadata handling and lifecycle. (a).**  **(Meta)data duples.** The metadata and the data form a (meta)data duple and are linked by a Data Identifier (DID – shown as vertical text), and each duple is considered an experiment. The metadata are stored in the blockchain, and the corresponding data within buckets inside the cloud storage. In the example, there are two buckets named *Project 1* and *Project* 2. **(b).** **(Meta)data lifecycle.** data and metadata processing within the (meta)data duple follow distinct procedures. *Upload*: The metadata are first validated by the blockchain quality control, if this passes validation by consensus, the metadata are added to the ledger, and the corresponding data are stored in the cloud. *Download*: In this current configuration, as only one channel is used, only one ledger is shared and so all users who have permission to access the channel are able to access the metadata, but further authentication is needed to retrieve the matching data. To keep track of data changes, a snapshot of the upload is performed by the version control software.

Metadata searching and data download can only be performed by peers within a consortium. When searching the ledger, the system will only return the metadata results for which the peer has read permissions (i.e., specified by their **channel** membership). Similarly, when a user attempts to download the data associated with selected metadata hit, a verification step (username and password) is performed to ensure access permissions. In our demo version of GADDS the metadata and data permissions are identical.

Metadata can also be modified in experiments, but corresponding data cannot be changed. When a request is made to modify an experiment, a new (meta)data duple is created in which the experiment name and identifier are maintained from the original entry. This new metadata will then be verified by consensus and, if successful, will be incorporated into a new block. Thus, the new metadata will point to the original data object. Each modification creates a snapshot in the Version control, so a history of all the modifications is kept in a history log, which is saved in the peer’s personal hard drive and in the cloud storage. If new data is created, a new duple will also be created.

1. **Blockchain**
   1. **Metadata validation steps**

Hyperledger Fabric implements multiple node types to provide various functions: the peers (or endorser, validator and committer (EVC) nodes – see below) that participate in metadata validation and storage; the certificate authorities that are responsible for permissions; and one ordering node within each organization that is in charge of ordering the metadata prior to appending to the ledger. For simplicity, the example GADDS platform has been configured to disable the certificate authorities’ nodes and generate the permissions beforehand.

The metadata validation mechanism starts when a user submits (meta)data through the interface and ends when the metadata has been stored in the ledger. The whole process is shown schematically in *Supplementary Figure 2*. The following steps occur during the metadata quality control process:

Step 1.1: The web interface sends a request to the EVC nodes to start the quality control process.

Step 1.2: The web interface packages and signs the metadata as a transaction and sends it to the EVC nodes.

Step 2.1: Each EVC node performs an endorsement check of the credentials of the Interface.

Step 2.2: At the same time, each EVC node performs a validation check of the metadata by comparing against a reference template.

Step 2.3: Each EVC node sends their endorsement (consensus) response to the Orderer node in their organization.

Step 2.4: Steps 1.1 to 2.3 are repeated for several submitted metadata transactions.

Step 3: The Orderer gathers several transactions, and orders and packages them into a block (this will also include transactions that were not endorsed).

Step 4: The Orderer sends the assembled block to all EVC nodes.

Step 5: The EVC nodes validate the order of each transaction within the block by consensus. This step is necessary because only the EVC nodes (not the Orderer) are trusted. This also enforces the decentralisation philosophy

Step 6: Each transaction within the block is updated with the result of the validation.

Step 7: Each peer adds the block to its copy of the ledger.

(This means that a block may contain one or more invalid metadata entries, but these are filtered out when a user queries the ledger).


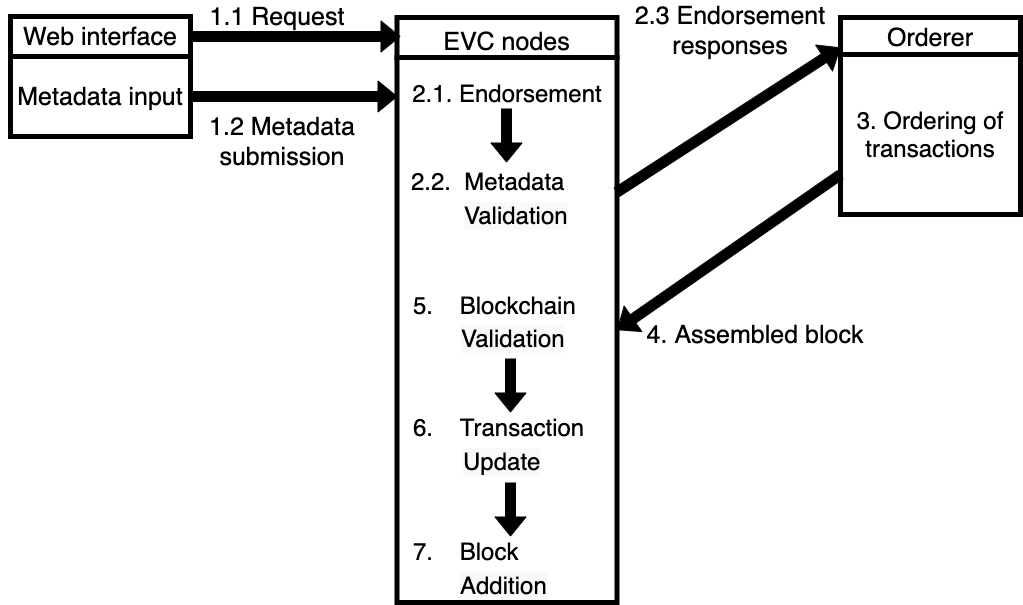


**Supplementary Figure 2.** **Metadata validation flow.** Schematic showing the steps taking place during the metadata quality control process between the web interface, the EVC nodes and the Orderer. The web interface interacts with the user where the metadata is input. Then the EVC nodes execute a series of steps to endorse submitted metadata and validate it. The function of the Orderer is to order and package metadata into blocks. The ordering within the blocks is then validated by the EVC nodes, if the validation is successful the block is appended to the ledger.

- 1. **Channels**

When executing a transaction, it is necessary to specify both the peers (EVC nodes) and the channel on which the transaction will be executed. Channels provide a completely separate communication layer between participants to maintain (meta)data and communication privacy, thus each channel has a unique ledger. In the GADDS example presented here, we have defined the EVC nodes to be within a single channel in single consortium, thus the GADDS example contains a single ledger.

- 1. **Certificates**

Hyperledger Fabric accommodates two specialised node types called the Membership service provider (MSP) node and the Certificate authority (CA) nodes. MSP nodes actively manage identities, CA nodes issue certificates to grant permission to participants. Within GADDS, we predefine identities upon start-up via certificates as this further support decentralisation by removing dependency on dedicated certification agents.

1. **Data storage**

The GADDS platform uses a cloud architecture where data is split, replicated, and stored across multiple devices. *Supplementary Figure 3* shows and example of how a datafile is handled by the cloud.

To split files the GADDS platform uses Minio Erasure code^[[1]](#footnote-1)^. In practice, it is recommended that the distributed deployment consists of homogenous nodes and storage capacity should reflect the size of the files being stored.


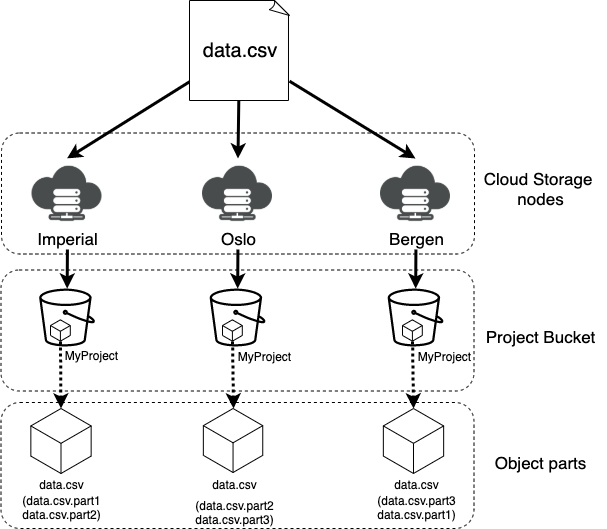


**Supplementary Figure 3. Schematic of file replication in the MinIO cloud storage system.** The example shows how a single uploaded file is split and replicated among all storage nodes. The Supplementary Figure shows how a file is stored in the form of an object and assigned to a bucket.

1. **Version control**

DIVECO is capable of recording changes made to (meta)data entries, so that submissions that have been already validated can be modified. When submitting a change to the (meta)data through the web interface, the process of validation is initiated as if it was a new entry. If the validation is successful a new block of metadata is created. The older version of the metadata is retained so, consistent with other version control software such as Git(Git, 2021), this makes it possible to “go back in time” to retrieve a specific version of that metadata. However, the corresponding data cannot be modified, thus both versions of the metadata point to the same data*.*

When first submitting and validating the (meta)data duple, the Hyperledger Fabric creates a unique MID. At the same time, DIVECO creates a DID that uniquely identifies the data associated with the metadata, see Supplementary Figure 6. If there is a request to change the metadata, a new MID will be generated but the DID will remain the same and a new duple will be generated. This process is shown *Supplementary Figure 4.*


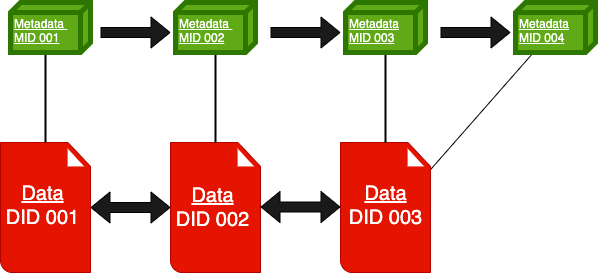


**Supplementary Figure 4**. **Schematic of the Distributed Version Control (DIVECO) system used in the GADDS platform.** Each metadata entry in the ledger has a unique Metadata Identifier (MID), e.g. MID001, which points to its corresponding data through a unique Data Identifier (DID), e.g. DID001. A change to the metadata, requires new metadata entry to be validated by consensus. The new MID (e.g., MID004) will then point to the original data.

1. **Docker**

We use Docker containers and Swarm technology for its ease of use and flexibility. In Docker, a container acts as an independent software package and this ensures that the platform can work uniformly across machines with different hardware or operating systems. Additionally, the Docker Swarm simplifies the resource management (of the containers) across multiple machines, to ensure they are handled efficiently. This is especially relevant when deploying packaged code across multiple computers in a test environment, where code updates may be required. In the current GADDS platform implementation, we have simplified the allocation of containers, such that an organization accommodates two EVC nodes, an Orderer, a Web Interface and a Cloud Storage. Nevertheless, Docker flexibility allows different configurations where containers can be allocated in different hardware.

1. **Security**

The GADDS is intended to be deployed in private networks with known participants, such as University networks, thus security concerns are more limited compared to a public blockchain network. A node can act maliciously when its metadata validation algorithm has been tampered with, causing it to behave differently in the consensus algorithm. However, it will require that more than half of the total nodes in the consortium be compromised for invalid metadata to be appended to the blockchain. Even in a simple case of a single consortium with three organizations, (as in the example presented in this paper) this will require that at least two organizations, which are in two different security environments (i.e., behind two different Universities firewalls) be compromised. At the same time, the tampering of certificates used during the endorsement (see next paragraph), could also result in a security issue, but this will also require that the majority of nodes be compromised as there is a system of verification of certificates among peers.

1. **User Interface**

The users (i.e. researchers) interact with the GADDS platform through a web browser interface. The web interface communicates with a Nginx server running in a Docker container. This container also supports the version control DIVECO and it is allocated, along with the Orderer container, in the organizations’ hardware installation. In order to access the user interface, the users need to specify either their Organization’s IP or domain name. Thus, the organizations’ nodes need a suitable network and firewall configuration to permit access. In the GADDS platform example, access to the web interface is only possible through the Eduroam internet service, or through institution Virtual Private Networks, so public access is not possible.

The user interface has an upload and a download web page presented as forms. The upload form, see *Supplementary Figure 5a*, requires entries specifying blockchain configuration (i.e., ledger), user authentication and metadata; a single submission button sends the form for validation, if successful the GADDS platform allows data upload into the cloud. The download form, see *Supplementary Figure 5,* also requires specification of ledger, user authentication and a search string; the corresponding metadata will be shown and a data download button allows access to all the corresponding data.


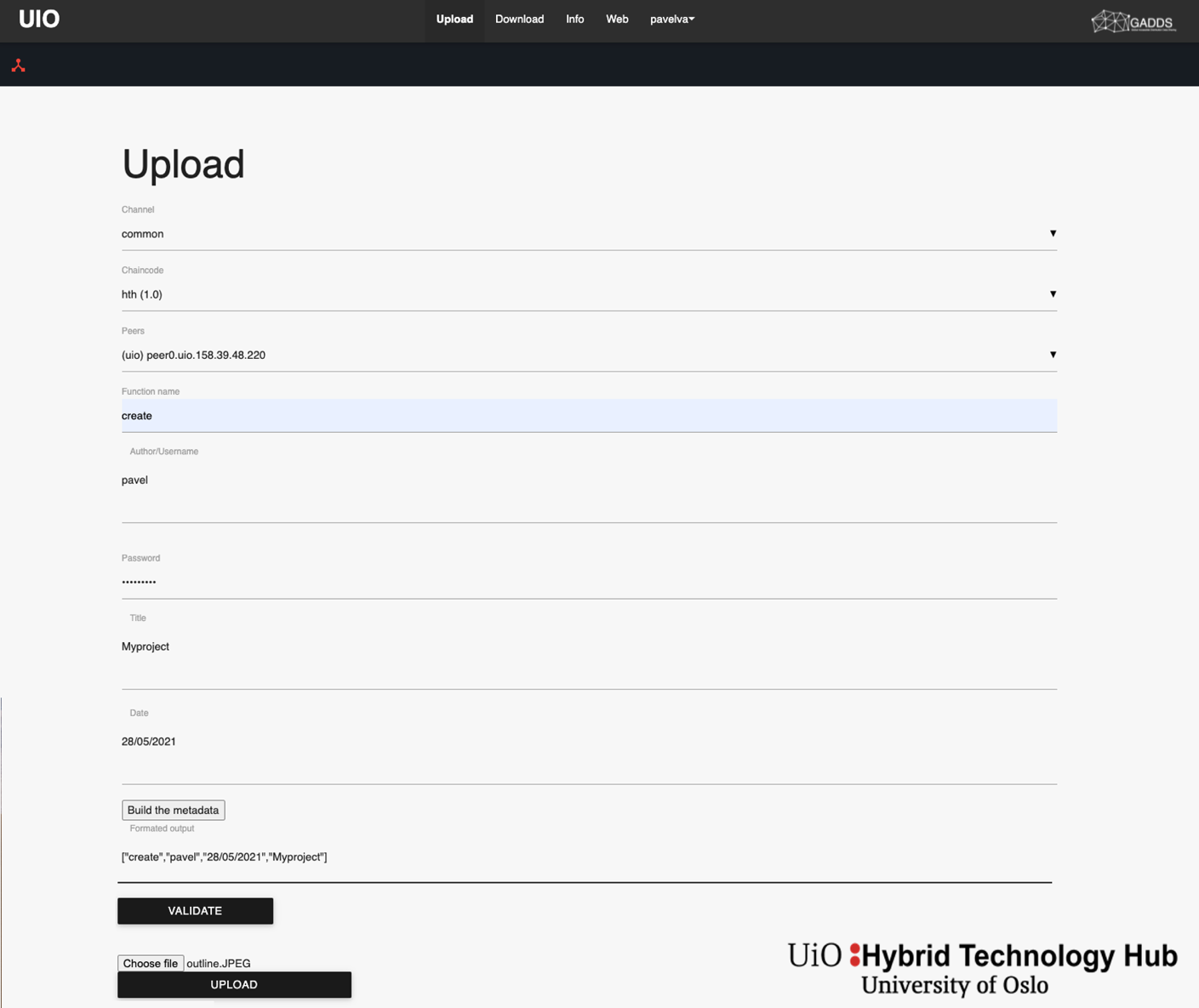


**Supplementary Figure 5.** **User interface.** The interaction with the GADDS platform is via web browser, where users can *(a)* upload and *(b)* search and download. The IP or the domain name of a suitable organization network (firewall and port) is needed to access the web page.

1. **Tables**

**Supplementary Table 1.** **Structural metadata and data used in Phase 1 (Tissue Engineering).**

| **Metadata** | **Data** |
| --- | --- |
| Cell Type (e.g. HEK293) | Cell concentration/Supplier/Passage number |
| ECM Composition (e.g., Collagen) | ECM concentration/Supplier |
| Outer Hydrogel Solution (e.g., Sodium Alginate) | Hydrogel concentration/Supplier |
| Cross Linking Solution (e.g., CaCl_2_) | Cross Linking Solution/Supplier |
| Nozzle Type (e.g., Single) | Flow rates |
|  | Nozzle inner diameter |

**Supplementary Table 2. Structural metadata and data used in Phase 2 (Fibre Measurement).**

| **Metadata** | **Data** |
| --- | --- |
| Number of measurements (e.g., 3) | Inner/Outer Diameters |
| Number of images (e.g., 5) | Image File |
| Image Resolution (e.g., 100 dpi) |  |

1. <https://docs.min.io/minio/baremetal/concepts/erasure-coding.html>, Last access: 05.04.2022. [↑](#footnote-ref-1)
